# Effect of GnRH agonist (deslorelin) on reproductive activity in captive female veiled chameleons (*Chamaeleo calyptratus*)

**DOI:** 10.17221/31/2023-VETMED

**Published:** 2023-07-27

**Authors:** Eva Cermakova, Zora Knotkova, Damian Boruvka, Misa Skoric, Zdenek Knotek

**Affiliations:** ^1^Avian and Exotic Animal Clinic, Faculty of Veterinary Medicine, University of Veterinary Sciences Brno, Brno, Czech Republic; ^2^Department of Pathological Morphology and Parasitology, Faculty of Veterinary Medicine, University of Veterinary Sciences Brno, Brno, Czech Republic

**Keywords:** female reptiles, ovarian follicles, reproductive medicine

## Abstract

Eighteen 5 months old veiled chameleon females (*Chamaeleo calyptratus*) were used in the study. Seven females received subcutaneous implants with gonadotropin-releasing hormone agonist (GnRH) deslorelin acetate whereas eleven females were used as control animals without any implants. Females were kept in five terraria, in groups of four females (in 3 terraria) and groups of three females (in 2 terraria), respectively. A minimum of one female with GnRH implants was present in each terrarium. They were kept under standard husbandry conditions. Females of both groups (females with GnRH and controls, respectively) were monitored for three years. No differences between females with GnRH implants and females without GnRH implants were observed with respect to the presence of large ovarian follicles, number of eggs/female, or number of clutches/female. GnRH implants did not prevent spontaneous reproductive activity in any of the 7 females with implants. Ovariectomy was performed in 8 females (4 females with GnRH implants and 4 control females). Ten females (3 females with GnRH implants and 7 control females) had to be euthanised. In 17 of 18 female veiled chameleons of this study histologic examination revealed heterophilic granulomatous oophoritis. The use of GnRH agonist implants did not prove to be an appropriate method for the control of reproductive function in captive female veiled chameleons.

The most common problems associated with pet lizard breeding include complications with incomplete ovulation (preovulatory follicle syndrome – POFS) or egg binding (post-ovulatory egg stasis – POES) (Barten 2000; Blazquez et al. 2000; Barten 2006; Dorrestein et al. 2007; Melidone et al. 2008; Pimm 2013). The most commonly affected species are green iguanas (*Iguana iguana*), veiled chameleons (*Chamaeleo calyptratus*), leopard geckos (*Eublepharis macularius*), and inland bearded dragons (*Pogona vitticeps*). Females that develop large ovarian follicles without contact with a male are at risk of ovulatory failure with subsequent follicular atresia and damage to the health and subsequent loss of a valuable animal is high.

In clinical practice, cases of spontaneous ovulation have been reported, but the frequency of such cases has not yet been reliably assessed either in captive female lizards or in female lizards in the wild. At approximately 6–7 months of age and at a weight that often does not even reach 100 g, many female veiled chameleons are presented to veterinary clinics with active ovarian follicles (authors’ clinical experience for more than last 20 years). The solution is to mate them with a male or perform a prophylactic ovariectomy. This is a standard surgical procedure and the methodology is well established (Knotek et al. 2017; Knotek and Wilkinson 2018; Stahl 2019). However, it inevitably results in the loss of the reproductive potential of the individual. Some studies focused on the possibility to regulate ovarian activity in female reptiles with hormonal implants [tamoxifen, indomethacin (DeNardo and Helminski 2000); gonadotropin-releasing hormone agonist– GnRH (Knotek et al. 2009; Kneidinger et al. 2010; Cermakova et al. 2019; Bardi et al. 2021)]. In one of our previous studies, we documented that GnRH implants did not regulate ovarian activity in captive leopard geckos (Cermakova et al. 2019). On the other side, GnRH implants influenced significantly the ovarian activity in captive female green iguanas [(evaluated with control of reproductive activity, control of the presence of ovarian follicles, and control of oestrogens and progesterone in plasma (Knotek et al. 2009; Kneidinger 2010)]. Also recently published results of the experimental use of GnRH implants in sliders (*Trachemys scripta*) suggested the possibility of regulating the ovarian activity in female reptiles with GnRH implants (Bardi et al. 2021). The aim of this study was to determine if GnRH implants could be used for the regulation of the reproductive activity in captive young female veiled chameleons.

## MATERIAL AND METHODS

### Animals

Eighteen three-month-old female veiled chameleons (*Chamaeleo calyptratus*) were housed and managed with the agreement of the Branch Commission for Animal Welfare of the Ministry of Agriculture of the Czech Republic (Accreditation No. 45620/2008-17210, 45620/10001). After the age of two months, females were divided into two categories (7 experimental females and 11 controls). Prior to the next procedure, each animal was given a complete physical examination, including a recording of the body weight (Table 1), and checked for the presence of intestinal parasites (only female chameleons without the presence of parasites, especially of coccidia in their faecal samples, were used in this study).

**Table 1:** Body weight of female veiled chameleons at the age of 5 months

Body weight (g)			
Control group (*n* = 11)		GnRH group (*n* = 7)	
71.0		74.0	
75.5		62.8	
57.4		72.7	
78.0		84.0	
84.0		57.0	
65.6		62.6	
83.0		74.0	
66.0		–	
73.0		–	
76.7		–	
82.5		–	
Mean	73.90	Mean	69.60
Median	75.50	Median	72.70
SD	8.36	SD	9.22

GnRH = gonadotropin-releasing hormone

### Implant administration

Implants with deslorelin (Suprelorin^®^; 4.7 mg; Virbac Animal Health, Carros, France) were administered to 7 females in the period of minimal ovarian activity [absence of large ovarian follicles was documented with radiography and plasma chemistry (Knotek et al. 2008)]. Females were fasted for 24 h before the anaesthesia. Meloxicam (1 mg/kg, Metacam 2 mg/ml; Boehringer Ingelheim Vetmedica GmbH, Ingelheim am Rhein, Germany) was administered intramuscularly in the left front leg. Tramadol (10 mg/kg, Tramal 50 mg/ml; STADA Arzneimittel, Bad Vilbel, Germany) was administered intramuscularly in the right front leg. Forty-five minutes later alfaxalone (5 mg/kg, Alfaxan 10 mg/ml; Jurox, Crawley, UK) was administered intravenously to the ventral tail vein. Implants were administered subcutaneously on the right body side. The recovery was uneventful. Fifteen to twenty minutes after alfaxalone administration all female veiled chameleons were active, without any complications during recovery. Eleven females from the second group were used as control animals.

The female veiled chameleons were kept in five terraria, in groups of four females in the same terrarium (3 terraria) or in groups of three females in the same terrarium (2 terraria), respectively. In each terrarium minimum of one female with GnRH was present. Females were kept under standard husbandry conditions in terraria (75 cm × 88 cm × 75 cm each) with a 12-h/12-h day/night cycle provided by 60 W incandescent bulbs, and basking provided by UV lamps (Repti-Glo 10.0; Hagen Corp., Mansfield, USA). Temperatures in the terraria ranged from 24 °C to 35 °C with night drop, and air humidity from 60% to 80%. Each terrarium was equipped with wood branches and artificial vegetation (plastic leaves), and clean paper was present on the bottom of terrarium. Clean water was offered *ad libitum* in plastic dishes. Every day fogging was used to keep the optimal humidity inside the terraria. Food (crickets) with mineral and vitamin powder (Nekton-MSA plv., Nekton Products, Dammfield, Germany) was offered every second day. Females of both groups (GnRH and controls, respectively) were monitored for three years with respect to the presence of large ovarian follicles, number of eggs/clutch/female, or number of clutches/female.

### Surgeries or necropsies performed on female veiled chameleons after the three-year study

In 8 females (4 females with GnRH implants and 4 control females), bilateral ovariectomy was performed to solve severe difficulties associated with the presence of follicles (weight change, abdominal distension, discolouration, anorexia, and incipient apathy). Ten females (3 females with GnRH implants and 7 control females) were left without surgical intervention, but due to the chronic (3–4 weeks) progression of their clinical condition they had to be eventually euthanised with ketamine (20 mg/kg i.m., Narkamon 50 mg/ml; Bioveta a.s., Ivanovice na Hané, Czech Republic), medetomidine (0.1 mg/kg i.m.; Cepetor KH 1 mg/ml; CP Pharma, Burgdorf, Germany) and a mixture of embutramidum, mebezonii iodidum and tetracain hydrochloridum (0.4 ml, i.v., T61; Intervet International GmbH, Unterschleisheim, Germany). Ovaries collected during the ovariectomy or post-mortem (in euthanised animals) were fixed in 10% formalin solution, for 24 hours. Standard protocol for histology with staining the ovarian tissues with haematoxylin-eosin (H&E) and PAS was performed at the Department of Pathological Morphology and Parasitology, Faculty of Veterinary Medicine, VETUNI Brno, Czech Republic.

## RESULTS

Implant administration was not followed by any postoperative complications or health problems at any point in the period of the whole study. No significant differences were observed in groups (females with GnRH implants, control females) with respect to the presence of large ovarian follicles, the mean number of eggs/clutch/female (50 ± 23 and 48 ± 18, respectively), or the mean number of clutches/female (5.8 ± 1.0 and 6.2 ± 1.3, respectively). GnRH did not prevent reproductive activity (the presence of large ovarian follicles followed by the presence of eggs) in any of the 7 females with implants.

In 17 of 18 female veiled chameleons of this study histologic examination revealed heterophilic granulomatous oophoritis. In one female with GnRH implant, the necropsy revealed active ovarian tissue with follicles (Table 2).

**Table 2:** Histology of ovarian samples collected during ovariectomy or necropsy from 18 female veiled chameleons

Females	Group	Necropsy (n) Ovariectomy (o)	Histology
1	control	n	heterophilic granulomatous oophoritis
2	control	n	heterophilic granulomatous oophoritis
3	GnRH	o	heterophilic granulomatous oophoritis
4	GnRH	o	heterophilic granulomatous oophoritis
5	control	n	heterophilic granulomatous oophoritis
6	GnRH	n	heterophilic granulomatous oophoritis
7	GnRH	n	heterophilic granulomatous oophoritis
8	GnRH	o	heterophilic granulomatous oophoritis
9	control	o	heterophilic granulomatous oophoritis
10	control	n	heterophilic granulomatous oophoritis
11	control	n	heterophilic granulomatous oophoritis
12	control	n	heterophilic granulomatous oophoritis
13	control	n	heterophilic granulomatous oophoritis
14	control	o	heterophilic granulomatous oophoritis
15	GnRH	o	heterophilic granulomatous oophoritis
16	GnRH	n	active ovarian tissue with presence of growing follicles
17	control	o	heterophilic granulomatous oophoritis
18	control	o	heterophilic granulomatous oophoritis

GnRH = gonadotropin-releasing hormone

## DISCUSSION

Deslorelin (Suprelorin^®^ 4.7 mg; Virbac Animal Health, Carros, France) was used in reptiles, specifically in a male bearded dragon to reduce his aggression towards the breeder (Rowland 2011). Deslorelin was also experimentally administered to female green iguanas, achieving suppression of follicular development and reduction of oestrogen and progesterone peaks to near basal levels in five of six females (Knotek et al. 2009; Kneidinger et al. 2010). No health complications were detected in any of the female lizards studied, and blood test results were also consistent with values found in healthy green iguanas. Standard reproductive activity was observed in three control females and two of them laid fertilised eggs.

No significant differences in the number of clutches between the female groups (deslorelin implants versus placebo implants) were found in a previous study with female leopard geckos. Deslorelin acetate implants did not interfere with reproductive activity in captive female leopard geckos and the use of GnRH agonist implants did not prove to be an appropriate method for control of reproductive function in captive female leopard geckos (Cermakova et al. 2019).

Recent studies in red-eared sliders suggest that GnRH implants could at least partially affect reproductive activity in terrapins (Bardi et al. 2021); however, other long-term observations have reached a less optimistic conclusion (our yet-to-be-published results). The results of research studying the findings in the ovaries of female veiled chameleons with GnRH implants have not yet been published. In our study of female veiled chameleons, where the implant with deslorelin was inserted into the subcutaneous tissue of 7 female lizards, no reduction in reproductive activity was achieved. All female veiled chameleons in the study population exhibited natural reproductive activity as reported for this species (Kummrow et al. 2010a; Kummrow et al. 2010b).

Check-ups showed that all female veiled chameleons were confirmed to have developed ovarian follicles and they repeatedly laid eggs. The subcutaneous implants were removed postmortem. It was found that the implants were slightly encapsulated in a fine layer of collagen fibres. The same result was observed in another study in a group of veiled chameleons (Kummrow 2009) where intracoelomic tamoxifen implants were encapsulated by granulation tissue. Even in this case, the implants had no significant effect on the regulation of the reproductive cycle of female veiled chameleons.

In conclusion, it could be stated that results of our study suggest that GnRH implants did not prevent spontaneous reproductive activity in captive female veiled chameleons. Although there is a large body of information on the physiology of the reproductive cycle in reptiles, including monitoring of hormonal activity (Gouder et al. 1979; Jones et al. 1979; Jones and Guillette 1982; Jones et al. 1988; Mendez De La Cruz et al. 1993; Shanbhag et al. 2001; Desantis et al. 1998; Amey and Whittier 2000; Edwards and Jones 2001; Cuadrado et al. 2002; Gardner and Barrows 2010), information on the possibility of influencing the reproductive cycle of reptiles using hormonal preparations is limited. Some results are even contradictory (Licht 1970; Callard et al. 1972; Callard and Doolittle 1973; Burns and Richards 1974; Licht and Crews 1975; Licht et al. 1984; Jones et al. 1990; Millar 2003; Herbert and Trigg 2005; Schneider et al. 2006).

The results of experiments attempting to influence the hormonal activity of reptile testicles have also been negative (Kirchgessner et al. 2009; Mason et al. 2021). Further research in this direction is therefore still relevant.
